# Peroneus brevis split tear – A challenging diagnosis: A pictorial review of magnetic resonance and ultrasound imaging – Part 2: Imaging with magnetic resonance and ultrasound

**DOI:** 10.1016/j.ejro.2024.100627

**Published:** 2024-12-22

**Authors:** Katarzyna Bokwa-Dąbrowska, Dan Mocanu, Isaac Romanus, Rafał Zych, Michael Huuskonen, Pawel Szaro

**Affiliations:** a Department of Radiology, Institute of Clinical Sciences, Sahlgrenska Academy, University of Gothenburg, Gothenburg, Sweden; b Department of Musculoskeletal Radiology, Sahlgrenska University Hospital, Gothenburg, Sweden; c Department of Clinical and Descriptive Anatomy, Medical University of Warsaw, Poland

**Keywords:** Tear, Rupture, Tendon, Ultrasound, Magnetic resonance imaging

## Abstract

Peroneal tendon pathology is common among physically active individuals, with tenosynovitis, tendon subluxation, split tears and rupture. However, diagnosing these conditions, particularly peroneus brevis split tears, is clinically and radiologically challenging. Magnetic resonance imaging (MRI) and ultrasound (US) can sometimes miss split tears. A significant portion of peroneus split tears develops on a background of tendinopathy. The presence of tenosynovitis, changes in tendon shape, and multiple subtendons can indicate a complete multifragmenting split tear. A defect on the surface of the tendon may indicate a partial-thickness split tear, commonly referred to as the "cleft sign." Peroneus subluxation is particularly likely when the superior peroneal retinaculum is torn. Given the subtlety of clinical symptoms, radiological evaluation is essential. Dynamic US assessment is especially valuable for detecting instability and split tears. This pictorial review presents the imaging spectrum of the most common pathologies of the peroneus brevis tendon on US and MRI.

## Highlights

• Outline changes and peroneus brevis thinning are key indicators of split rupture.
• Tenosynovitis is common but not a specific hallmark of peroneal tendon pathology.
• "Tear" refers to longitudinal split, while "rupture" refers transverse discontinuity.
• In radiological reports of peroneus split the length and location should be provided.

## 1 Risk factors and incidence

Risk factors for peroneus brevis split rupture remain unclear. Clinically significant anatomical variants are discussed in Part 1 of the review. Contributing factors include a low-lying peroneus muscle belly, accessory muscles like the peroneus quartus, retromalleolar groove abnormalities, and chronic ankle instability. A high body mass index (BMI), eccentric inversion, and rapid plantar flexion in sports increase the risk of ankle sprains and peroneus split tears, especially in athletes [1], [2]. Rheumatoid arthritis and diabetes also increase the risk of peroneus brevis split tear [3].

**Fig. 1:**
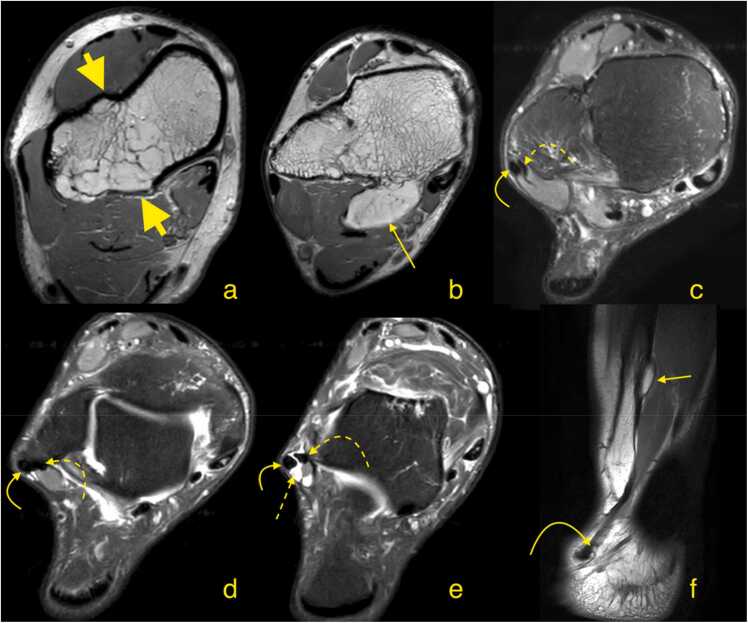
Patient with multiple hereditary exostosis. Magnetic resonance imaging (a and b - transverse section, proton density weighted; c, d, e - oblique sections; c is the highest level, e is the lowest level, proton density weighted with spectral attenuated inversion recovery; f - T1-weighted, sagittal section through the fibula). The examination shows multiple exostoses with ankylosis between the distal tibia and fibula (a - short thick arrows) as well as exostosis impacting the muscles (long narrow arrows). The peroneus longus (curved arrow) and peroneus brevis (curved dashed arrow) are displaced laterally from the deformed lateral malleolus. Effusion is present in the common synovial sheath (straight dashed arrow).

Bone-related factors include retromalleolar groove irregularities [4], [5], concave grooves, and prominent ridges causing instability [6], [7]. Congenital hindfoot deformities and bone anomalies like exostosis (Fig. 1) also contribute to tendon tears [8], [9].

A low-lying muscle belly may double the risk [10], [11]. Post-traumatic superior peroneal retinaculum instability leads to tendon subluxation and split tears, especially after ankle sprains [12], [13].

While obesity is linked to tendon tears [14], studies have found no significant BMI differences in split tear cases, though higher BMI may accelerate tendinopathy [15].

No genetic studies exist for peroneus brevis splits, but COL5A1 variants reduce Achilles tendon injury risk [16], with tenascin and COL5A1 linked to Achilles tendinopathy [17].

High loads, collagen changes [18], reduced vascular supply, and inflammation (interleukin (IL)-1β, IL-6, tumor necrosis factor -α) heighten chronic tendinopathy risk [25], but most research focuses on the Achilles tendon [19].

## 2 Imaging diagnostic

Imaging of peroneus brevis pathology is challenging. X-ray and computed tomography (CT) can reveal the "fleck sign," which indicates superior peroneal avulsion [20]. Ultrasound (US) and magnetic resonance imaging (MRI) can detect most peroneal split tears. Both tendons curve beneath the lateral malleolus, causing US anisotropy, which can be minimized by adjusting the probe. In MRI, the tendons' oblique course can create a "magic angle" artifact, especially when collagen fibers are at a 55-degree angle to the magnetic field, and a short echo time is used [21], [22].

### 2.1 Diagnostic value of MRI and US

#### 2.1.1 Challenges in comparing studies

Comparing previous studies on the value of US and MRI in diagnosing peroneus brevis splits is challenging due to differing methodologies. The accuracy of MRI and US varies, with MRI sensitivity for peroneus brevis split tear detection ranging from 57 % to 100 % [23], [24] and US sensitivity from 88 % to 100 % [24], [25]. Although smaller, US-based studies consistently report high sensitivity and specificity [24], [25], [26], [27]. Expert radiologists used high-frequency probes and followed similar protocols across these studies. Two studies employed multiple blinded assessors to mitigate user dependency in tear grading [24], [27]. Technical advancements over time also affect comparisons; for example, Melville et al. reported 88 % sensitivity and 100 % specificity using high-resolution Doppler US, while Waitches in 1998, without Doppler US, reported 100 % sensitivity but only 67 % specificity [24], [27]. Both Melville and Grant highlight US's significance in detecting and differentiating tears from subluxation and tendinosis [24], [26].

#### 2.1.2 Magnetic resonance in peroneus brevis diagnostic

In our center, the MRI protocol for the ankle includes proton density (PD) in the transverse plane, T2-weighted Spectral Attenuated Inversion Recovery (SPAIR) in the sagittal plane, PD-weighted SPAIR in the coronal plane, oblique PD-weighted SPAIR, and sagittal T1-weighted images turbo spin echo. For postoperative cartilage or patients with suspected cartilage pathology, additional three-dimensional (3D) gradient echo (GRE) is included. GRE is valuable in ankle imaging due to its sensitivity to magnetic susceptibility effects, enabling the detection of hemosiderin deposits and blooming artifacts in conditions such as tenosynovial giant cell tumor, which may involve the peroneal tendon sheath or in the ankle joint [28]. GRE is also useful for identifying cartilage abnormalities, postoperative cartilage imaging and the detection of subtle intra-articular loose bodies. Significant benefits from ultra-high-field MRI (7 Tesla) compared to standard 3 Tesla imaging have been demonstrated, providing more precise visualization of structures using routine clinical sequences [29]. In our center ankle MRI is performed on 3 T machine.

There is no widely standardized protocol in use. Some centers use oblique PD-weighted oriented perpendicular to the peroneal tendons, allowing for transverse sectional imaging of the peroneal tendons. The value of this special section has been demonstrated in previous studies [30]. Targeted imaging focused on specific structures offer greater diagnostic value than purely anatomical planes [31], as widely applied in imaging the anterior cruciate ligament.

Ankle imaging is particularly challenging because many structures do not follow standard anatomical planes but are instead oriented obliquely. A potential solution to this problem is isotropic imaging (where voxel dimensions are equal in all three planes), which allows for multiplanar reconstruction [32].

#### 2.1.3 Future imaging

The unclear and often contradictory data in the existing literature regarding the effectiveness of MRI and US highlight the need for further studies comparing the efficacy of both modalities. Advances in imaging techniques may also help reduce the number of misdiagnosed cases. Novel sequences and dynamic MRI or high-resolution US, hold potential for improving patient outcomes.

## 3 Clinical symptoms

Peroneus brevis split tear symptoms are unclear, and clinical exams may not detect it. The split tear can result from chronic changes or more acute trauma often obscure symptoms. Peroneus split tear manifests as swelling and tenderness along the tendon pathway, particularly along the lateral wall of the calcaneus [33]. Pain frequently worsens during physical activity [34]. In some cases, observation of the patient's gait can reveal difficulty in weight-bearing on the affected limb, affecting proprioception and leading to improper weight distribution [35]. On clinical examination, the patient may experience pain during inversion stretching, resisted dorsiflexion, and both passive and active eversion. Additional clinical signs of peroneus brevis split tear include crepitus and cracking during dorsiflexion and plantarflexion [33].

## 4 Pathology

### 4.1 Pathophysiology

The exact pathophysiology of peroneus tendon split tears remains unclear however, they typically develop on a pre-existing tendinopathy [22], [34], [36]. These injuries may result from overuse, hypoxemia, tendon degeneration, instability, trauma. Split tears occur more frequently in the peroneus brevis tendon compared to the peroneus longus. The malleolar groove is the most common site for peroneus brevis tears, whereas peroneus longus tears are typically found in the cuboid notch [35], [37]. A commonly described injury mechanism involves chronic ankle instability and repetitive subluxation of peroneus brevis [38]. It is hypothesized that peroneus split tears develop through two main mechanisms: chronic overuse which is the most common cause, and less frequently, acute injury [33], [39].

### 4.2 Tenosynovitis

**Fig. 2:**
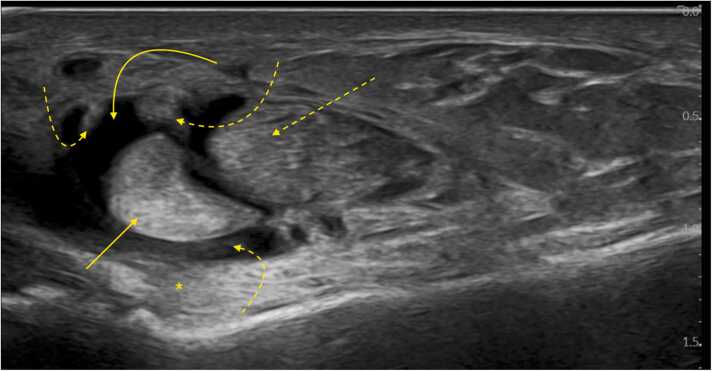
A patient with pain at and below the level of the lateral malleolus. Ultrasound, oblique section just below the level of the lateral malleolus, shows effusion in the common peroneal synovial sheath (curved arrow) and thickening of the synovial membrane (curved dashed arrows). The peroneus brevis (straight arrow) and peroneus longus (straight dashed arrow) tendons are intact but exhibit degenerative changes and moderate swelling. The calcaneofibular ligament is marked with an asterisk.

**Fig. 3:**
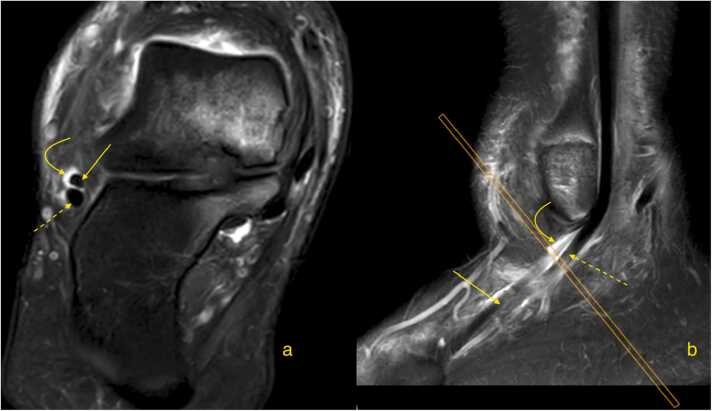
A patient with a recent ankle injury presenting with joint pain. Magnetic resonance imaging shows: (a) Proton density-weighted image with fat suppression in an oblique section (the level of the section is indicated on image b), and (b) Proton density-weighted image with fat suppression in a coronal section. Effusion in the common peroneal synovial sheath (curved arrow) is more prominent inferior to the lateral malleolus. The peroneus brevis (straight arrow) and peroneus longus (straight dashed arrow) tendons appear normal.

Effusion in the common peroneal synovial sheath and thickening of its synovial membrane coexists with approximately half of peroneus brevis split tears [36], [40]; however, detecting tenosynovitis is not specific to a split tear. Absence of fluid at the superior peroneal tunnel does not rule out its presence distally (Fig. 2, Fig. 3). The common peroneal sheath, which resembling a pair of pants with "legs" directed downward, can also accumulate fluid around the tendons distal to the peroneal tubercle. Fluid tends to accumulate more below the superior peroneal retinaculum due to its wider space. The sheath is filled with fluid, has a thickened synovial membrane, and Doppler imaging often shows hyperemia.

### 4.3 Tendinopathy

Tendinopathy includes tendinosis (tendon degeneration from chronic overuse) and tendinitis (inflammation like reaction from acute micro-tears). Peroneus brevis tendinopathy can lead to a peroneus brevis split tear, with most split tears having a degenerative origin [24], [40]. Imaging may show tendon thickening, disrupted fibrillar structure, decreased US echogenicity, and higher signal on MRI on PD and T2-weighted, often with coexisting tenosynovitis. Doppler frequently reveals increased blood flow in the tendon and synovium. There is no consensus on which anatomical variations may predispose to peroneus brevis tendinopathy (anatomical variations are discussed in the first part of our review), but retrospective studies show that over half of cases are secondary to ankle injuries, commonly involving ankle ligament damage [38], [41]. Malalignment of the fibula following an ankle injury can lead to peroneal tendon subluxation, peroneal tendinitis, and peroneus brevis split tear [41].

### 4.4 Nomenclature split tear vs. rupture

**Fig. 4:**
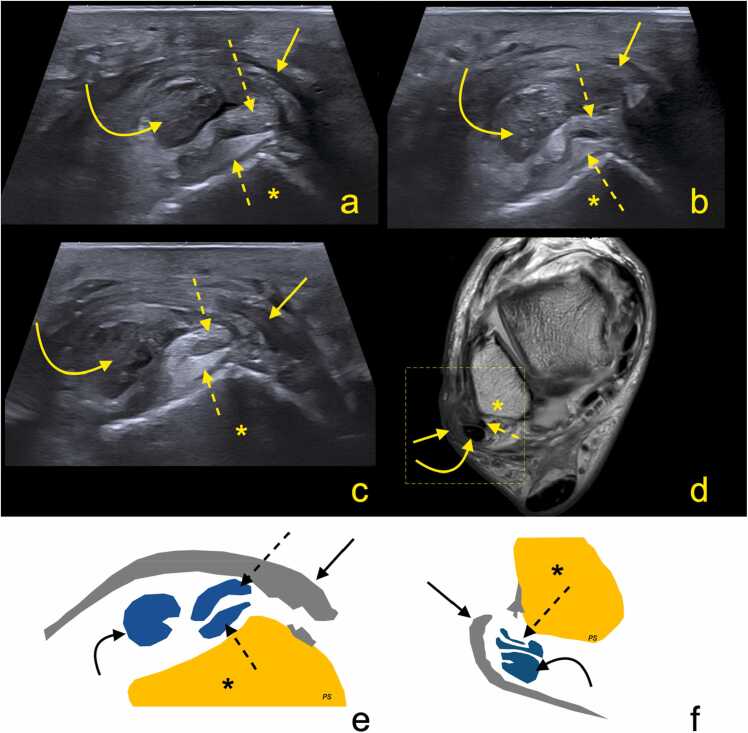
A patient with a history of an ankle sprain injury. Ultrasound (transverse sections a–c, with a being the most superior and c the most inferior section) and magnetic resonance imaging (proton density-weighted, transverse plane, d) reveal a peroneus brevis tendon split tear (dashed arrows). Schematic illustrations (e and f) provide simplified representations of the ultrasound image in segment (c) and a fragment of the MRI image makred by a dashed line in segment (d). The peroneus longus tendon (curved arrow) remains intact but shows tendinosis and a small, incomplete split along the anterolateral margin. Advanced tenosynovitis is present in the synovial sheaths. The superior peroneal retinaculum (straight arrow) is torn, leading to the dislocation of the peroneal tendons. The lateral malleolus is indicated by an asterisk.

The term "tear" generally refers to a longitudinal tear or partial rupture, while "rupture" typically indicates a complete tendon discontinuity, with the ends separated [42]. A split tear is considered complete if it affects the entire tendon thickness, and incomplete if it does not (Fig. 4).

### 4.5 Complete split tear

**Fig. 5:**
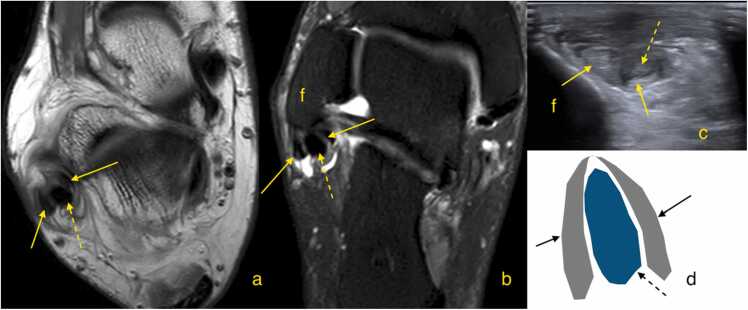
Patient with chronic ankle pain, approximately 9 months post-injury. Magnetic resonance imaging (a - proton density weighted, transverse section just below the lateral malleolus; b - proton density weighted with fat suppression, oblique section through the lateral malleolus). Ultrasonography (c) shows a transverse section through the lateral malleolus. The diagram (d) illustrates the "boomerang sign" on the transverse section. The dashed arrow indicates the peroneus longus, while the solid arrows point to two separate fragments of the peroneus brevis.

The radiological appearance of a complete tear varies with fragment displacement. Minimal or no displacement may show a "boomerang sign," as the subtendons envelop the peroneus longus (Fig. 5). Tendon caliber changes, such as focal thickening or thinning, should be confirmed on at least two sections in different planes [43]. With greater displacement, a split tear may appear as multiple subtendons. Subluxation of the split tendon is common, particularly with a superior peroneal retinaculum tear [38], [40], [44], [45].

**Fig. 6:**
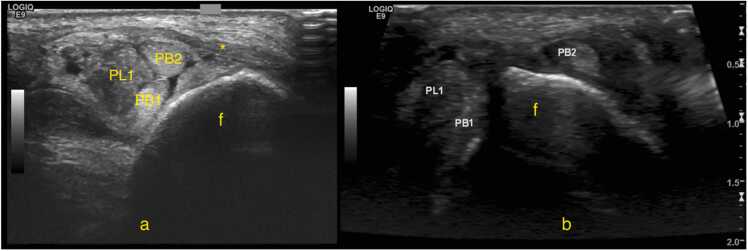
A patient with a history of an ankle sprain 7 months ago. Ultrasound, transverse sections at the level of the lateral malleolus. Section a is slightly higher than section b. Static examination (a), body weight-bearing examination (b). The study revealed a peroneus brevis split tear (PB1 and PB2 – two tendons’ fragments). During weight-bearing, one fragment (PB2) displaced to the lateral contour of the lateral malleolus (f), indicating instability due to damage to the superior peroneal retinaculum (asterisk). The peroneus longus (PL1) is in a normal position but shows signs of tendinosis.

**Fig. 7:**
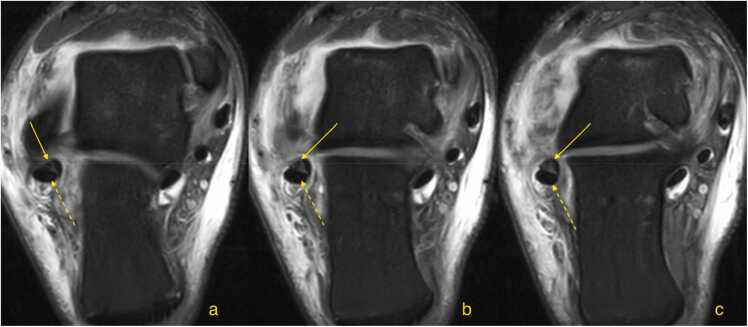
A patient with a recent ankle injury. Magnetic resonance imaging shows an acute peroneus brevis split tear (straight arrow) (a, b, c – oblique cross sections, proton density with fat suppression). The peroneus longus is normal (dashed arrow).

The split fragments of the peroneus brevis may be displaced by the peroneus longus [46]. A peroneus brevis split tear may also be associated with a superior retinaculum tear, which should be assessed dynamically (Fig. 6, Fig. 7). According to our observations, the sensitivity of US may be limited especially if the tear is small; therefore, we practice the dynamic and weight-bearing US examinations can help detect even small split tears.

### 4.6 Non-complete split tear

Non-complete split tears are characterized by a defect or focal thinning that affects only part of the tendon’s thickness [46]. On US or MRI, these tears typically present as areas of altered echogenicity or signal within the tendon, often starting from the deep fibers. Studies indicates that deviations from the normal flattened-oval shape to boomerang, arrowhead, or flame shapes may suggest a peroneus split tear [22], [47], [48].

**Fig. 8:**
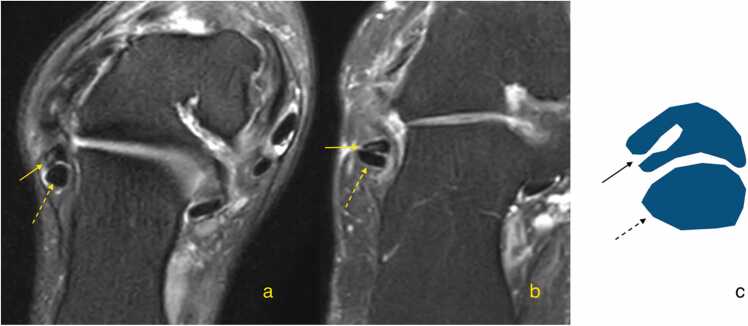
Cleft sign. Magnetic resonance imaging (a, b – proton density-weighted with fat suppression, oblique sections) shows a defect in the lateral outline of the peroneus brevis (straight arrow). The peroneus longus (dashed arrow) appears normal. A schematic corresponding to the cross-sections is shown in (c).

**Fig. 9:**
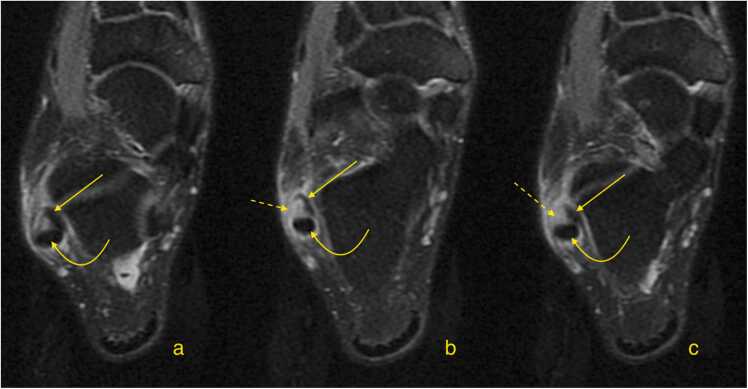
A patient with chronic lateral ankle pain. Magnetic resonance imaging (a-c proton density weighted with fat suppression) revealed a peroneus brevis split tear just below the level of the lateral malleolus (straight arrows) with *boomerang sign*, accompanied by tenosynovitis (dashed arrow). The peroneus longus tendon (curved straight arrow) appears normal.

**Fig. 10:**
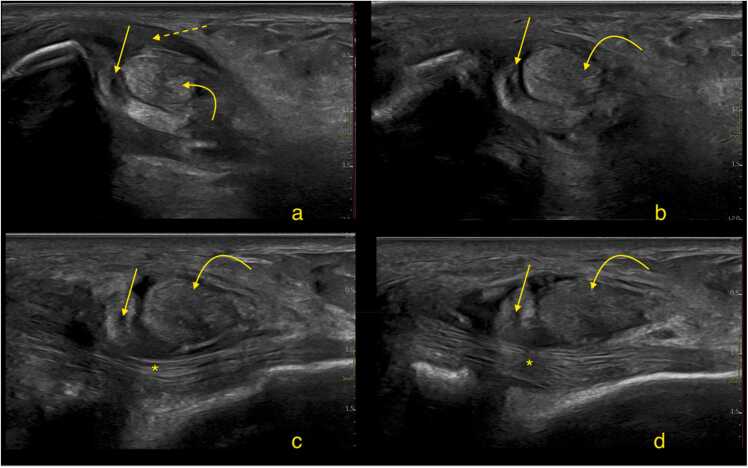
A runner presented with pain in the area posterior to the lateral malleolus. Ultrasound revealed a partial peroneus brevis split tear beginning at the level of the lateral malleolus with ultrasound-*cleft sign*. The tear extends inferiorly relative to the apex of the lateral malleolus (straight arrow). The peroneus longus is marked with a curved solid arrow, shows pathological changes, including swelling and abnormal echogenicity, consistent with advanced tendinopathy. The superior peroneal retinaculum is slightly thickened (dashed arrow). The calcaneofibular ligament is indicated by an asterisk. Cross-section a is the highest, while section d is the lowest. Sections a and b are transverse, while sections c and d are oriented obliquely, perpendicular to the course of the peroneal tendons.

The "cleft sign" is an defect affecting at least 50 % of the tendon’s diameter and breaching its surface, on MRI is seen as increased signal on proton density or T2-weighted images in two consecutive axial slices [49], Fig. 4, Fig. 8, Fig. 9. Based on our experience, such clefts can be relatively frequently identified in slightly thicker parts of the peroneus brevis tendon, more often on the lateral side and less frequently on the medial side (Fig. 10). However, there is a lack of studies on a large material to confirm our observations. Identifying the cleft sign on MRI can enhance a radiologist's diagnostic sensitivity for detecting peroneus brevis tears [49]. Peroneus brevis split tears are often accompanied by superior peroneal dysfunction or tear, tenosynovitis and bone marrow edema in the lateral malleolus.

#### 4.6.1 Radiological report in peroneus brevis split tear

The report should begin with confirming or excluding a split tear and, if present, noting its length and location relative to the lateral malleolus. Include any associated injuries to the ligaments and superior peroneal retinaculum. Occult tears may exist, so describe findings such as tenosynovitis or bone marrow edema, which can suggest a split tear even if not directly visible. Evaluate the muscle belly and rule out muscle atrophy.

#### 4.6.2 Classification of peroneus brevis split tear

**Fig. 11:**
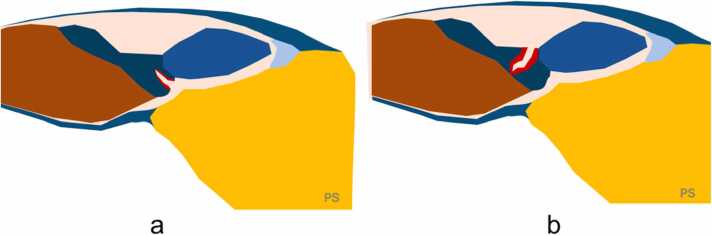
The classification, according to Krause and Brodsky, categorizes tears into two grades: grade 1 involves less than 50 % of the tendon thickness (a), while grade 2 involves more than 50 % of the tendon thickness (b).

**Fig. 12:**
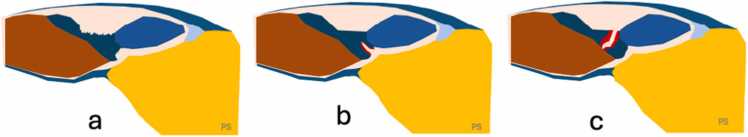
The classification according to Sobol, divides the tears into four grades: grade 1 shows only splaying of the tendon (a minor widening without a true split) (a); grade 2 is a partial-thickness split tear(b). Grades 3 and 4 represent full-thickness tears (c), with grade 3 tears measuring 1–2 cm in length and grade 4 tears exceeding 2 cm in length.

There is no one classification system for split tears that is widely used. In the literature there are two classifications of peroneus brevis split. Both classifications consider the percentage of tendon injury, Fig. 11, Fig. 12. The Krause and Brodsky classification [50] is a two-graded system (Fig. 11): Grade 1 involves less than 50 % of the tendon thickness, while Grade 2 involves more than 50 %. The Sobol classification [51] (Fig. 12) evaluates the extent of tendon thickness involvement and the longitudinal dimension of the tear. Grade 1 is only splaying of the tendon. Grade 2 involves a partial-thickness split tear. Grade 3 is a full-thickness tear measuring 1–2 cm in length; and Grade 4 is a full-thickness tear greater than 2 cm in length.

### 4.7 Rupture of peroneus brevis

**Fig. 13:**
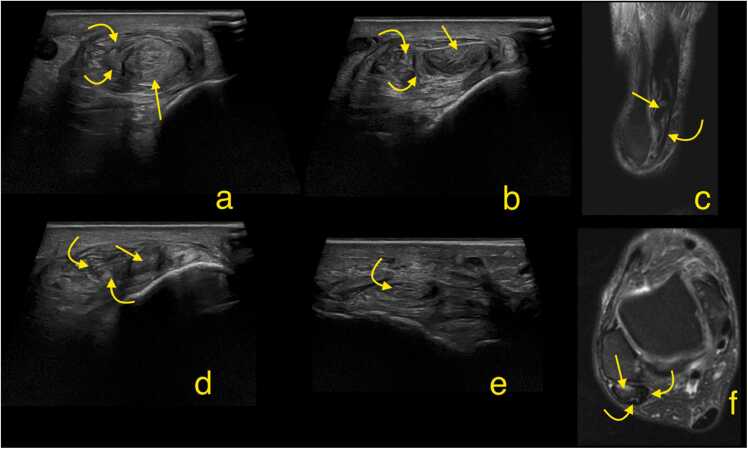
A patient with a history of an ankle sprain about one year ago, presenting with pain and limited range of motion in the ankle. Ultrasound (a, b, d, e) and magnetic resonance imaging (c and f) revealed a rupture of the peroneus brevis tendon with retraction of the tendon above the lateral malleolus (straight arrows). The peroneus longus tendon exhibits a split tear with small fragments (curved arrows) surrounding the stump of the peroneus brevis. Ultrasound transverse sections at the level of lateral malleolus (a and b). Magnetic resonance imaging: c- sagittal section, proton density-weighted with fat suppression showing fibula and peroneal tendons, d- slightly oblique section near the apex of the lateral malleolus, e- oblique section parallel and perpendicular to the peroneus longus tendon, f- transverse section, proton density-weighted with fat suppression at the level of lateral malleolus.

Complete ruptures are less frequent than split tears. Ruptures are characterized by a transverse orientated disruption in the continuity of the peroneus brevis tendon (Fig. 13). In chronic cases, significant diastasis between the tendon ends may develop, often accompanied by muscle belly atrophy, which can make reconstruction difficult or even impossible. Radiologists should assess the extent of diastasis between the tendon ends and the degree of fat atrophy in the muscle belly.

### 4.8 The instability of the peroneal tendons with a tear of the superior peroneal retinaculum

Posttraumatic superior peroneal retinaculum insufficiency, occurring after ankle injuries, can lead to peroneal tendon instability [47]. A thickened, loosened retinaculum, even with preserved continuity, can cause instability. Injury to the superior portion allows tendon dislocation during foot eversion, while damage to the inferior portion does not [48]. Oden classified superior peroneal retinaculum injuries into four types: type 1 involves a pouch at the fibular insertion, type 2 features a tear at the fibular insertion, type 3 involves a bony avulsion, and type 4 presents a posterior superior peroneal retinaculum tear, with types 1 and 3 being the most common [52].

Peroneal subluxations can affect both tendons and may be permanent or recurrent, triggered by dorsiflexion-eversion. US and MRI effectively detect superior peroneal retinaculum ruptures or peroneus brevis split tears, while dynamic US identifies non-permanent luxation. Some split tears and mild retinaculum thickening are detectable during weight-bearing US or ankle movement.

MRI and US can detect superior peroneal retinaculum thickening, rupture, and tenosynovitis. MRI may show bone marrow edema on the lateral malleolus, lateral subluxation of the peroneus longus, and a "fleck sign," indicating bony insertion of the superior peroneal retinaculum [20].

Anatomical variants' role in peroneal tendon instability is unclear, with one study finding no link between a low-lying peroneus brevis muscle belly and tendon dislocation [53].

Peroneal tendon stability may also depend on retromalleolar groove morphology and the fibrocartilaginous ridge, but larger studies are needed.

### 4.9 Internal subluxation

Intrasheath instability is a subtype of instability where the peroneal tendons switch positions during ankle movements without displacing onto the lateral malleolus and without damage to the superior peroneal retinaculum. It can occur with peroneal tendinopathy, and dynamic US reveals abnormal tendon movement while showing a normal retinaculum. Thickening of the retinaculum may indicate partial tears but is usually unrelated to instability [47].

**Fig. 14:**
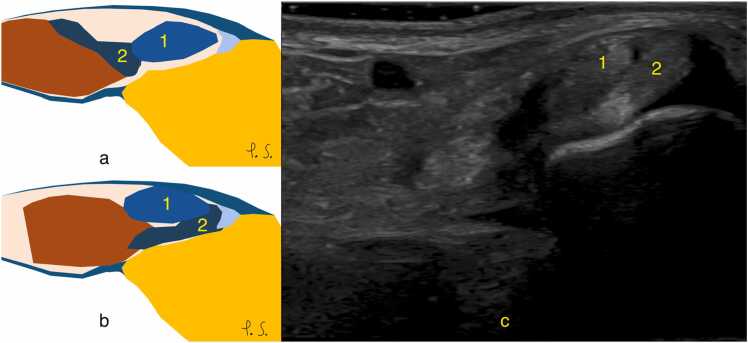
Instability of the peroneal tendons without a split or superior peroneal retinaculum tear. Normal anatomy is shown on a. Intra-sheath luxation (b and c): the peroneus brevis (2) is completely positioned under the peroneus longus (1) and is displaced laterally. Transverse cross-section (c) at the level of the lateral malleolus, ultrasound performed during dorsiflexion and eversion.

**Fig. 15:**
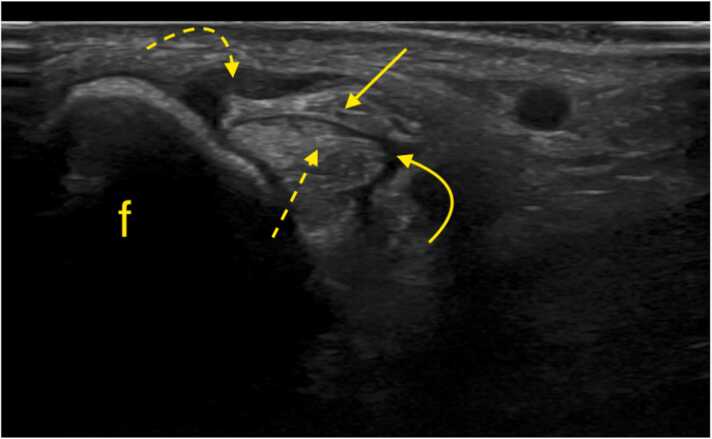
A patient with chronic pain posterior to the lateral malleolus. Ultrasound, transverse section at the level of the lateral malleolus (f), during dorsiflexion and eversion, revealed instability of the peroneal tendons. The peroneus brevis (solid arrow) is displaced above the peroneus longus (dashed arrow). The superior peroneal retinaculum appears slightly thickened (curved dashed arrow). Fluid is visible in the synovial sheath (curved solid arrow).

Raikin et al. classify two subtypes of intrasheath instability [5]. In type A, the peroneus longus and brevis remain intact but switch positions at the retromalleolar groove (Fig. 14 and Fig. 15). In type B, the peroneus brevis has a longitudinal split, allowing the peroneus longus to subluxate through it. Both types occur during active dorsiflexion or eversion, with the retinaculum intact.

US is essential for diagnosing peroneal instability because it may be missed on static MRI.

### 4.10 The role of radiological imaging and treatment perspectives

**Fig. 16:**
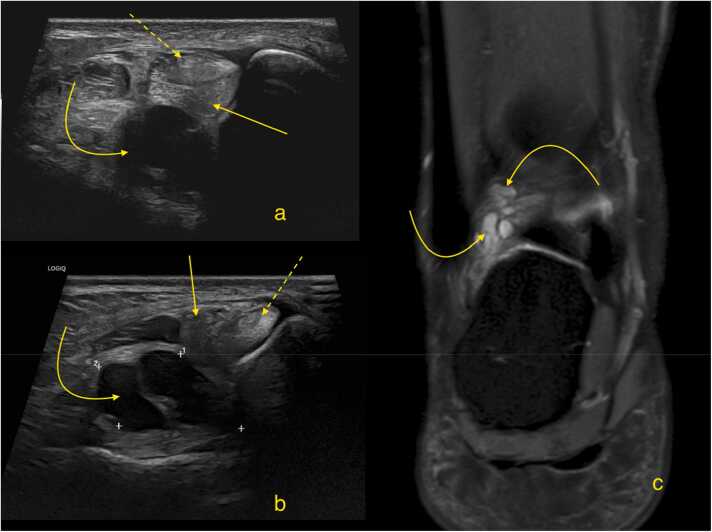
A patient with chronic pain behind the lateral malleolus. Ultrasound (a and b), transverse sections. Magnetic resonance imaging, coronal section (proton density-weighted with fat suppression, c). The peroneus brevis (straight arrow) and peroneus longus (straight dashed arrow) tendons show discrete signs of tendinopathy. A ganglion (curved arrow) is visible posterior to the peroneal tendons.

Imaging is used to find the source of the symptoms and for the preoperative planning. In the case of a peroneus brevis injury, it is essential to evaluate the extent of the tear by assessing the thickness of the tendon affected and the length of the tear. It is important to identify concomitant injuries, as discussed earlier. Differential diagnosis is also essential, because symptoms related to the peroneal tendons may arise from other causes, such as a ganglion displacing the tendons (Fig. 16).

Peroneus brevis split tear often resist conservative treatment, making surgical intervention necessary, particularly in cases involving tendon subluxation or split tear [54].

However, there are also asymptomatic peroneus brevis split tears which are find as incidental findings. The surgical treatment is typically reserved for symptomatic patients [40]. On imaging it is important to assess the localization and extent of the tear. Identifying accompanying injuries such as superior peroneal retinaculum tear, irregularities in the malleolar groove surface, and concomitant ligament tears can be valuable information for surgical planning.

Most authors recommend surgery if the split involves more than 50 % of the tendon. In our experience, assessing the extent of damage can be challenging on both US and MRI; therefore, a thorough evaluation in various foot positions may be helpful. If repair is not possible, an autograft (or, in some cases, an allograft) may be used. Due to superior biomechanical properties, grafting is preferred over tenodesis. When tenodesis is necessary, transferring the peroneus longus to peroneus brevis is generally the recommended approach, while transferring the peroneus brevis to the peroneus longus is not advised [42].

## 5 Conclusion

Diagnosing peroneus brevis split tears and instability remains a challenge for both clinicians and radiologists. Peroneus brevis pathologies can be visualized using both US and MRI. However, the value of MRI and US in the literature is inconsistent and sometimes contradictory, making comparison difficult. The presence of tenosynovitis is an important but non-specific indicator of potential peroneal tendon pathology. A split tear can be incomplete or complete, with two or multiple tendon fragments. Radiological signs, such as changes in the shape of the peroneus brevis, the "boomerang sign", "cleft sign" and alterations in tendon caliber, may suggest a split tear, although their diagnostic value remains unclear. Subluxation of the peroneal tendon is more likely when a tear of the superior peroneal retinaculum coexists. If initial imaging does not reveal abnormalities but clinical symptoms persist, follow-up imaging is recommended. Dynamic and weight-bearing assessments are crucial for detecting instability and certain split tears, which highlights the value of ultrasound in these cases.

## Ethics approval and consent to participate

No ethics approval is needed for this pictorial review.

## Consent for publication

Not applicable. The manuscript does not contain the data of individuals in any form.

## Funding

We acknowledge the financial support of the 10.13039/100010819Tornspiran Foundation, which enabled the preparation of this review article. This work was completed as part of our broader research activities supported by the foundation. The foundation had no influence on the content or conclusions presented in this article.

## CRediT authorship contribution statement

**Dan Mocanu:** Writing – review & editing, Writing – original draft, Visualization. **Rafal Zych:** Writing – review & editing, Writing – original draft, Visualization. **Katarzyna Bokwa-Dąbrowska:** Writing – review & editing, Writing – original draft, Visualization, Methodology, Conceptualization. **Isaac Romanous:** Writing – review & editing, Writing – original draft. **Michael Huuskonen:** Writing – original draft, Visualization. **Pawel Szaro:** Writing – review & editing, Writing – original draft, Visualization, Supervision, Conceptualization.

## Authors' contributions

**PS** conceived the idea of this review. **KBD, RZ, DM, IR** and **PS** contribute the first draft of the manuscript. **KBD** and **PS** selected appropriate figures and prepared figures. All authors read and approved the final manuscript.

## Declaration of Generative AI and AI-assisted technologies in the writing process

Nothing to disclose.

## Declaration of Competing Interest

The authors declare that they have no known competing financial interests or personal relationships that could have appeared to influence the work reported in this paper.

## Data Availability

Yes.
